# Characteristics of rhizosphere and bulk soil microbial communities in pear trees

**DOI:** 10.7717/peerj.20627

**Published:** 2026-01-15

**Authors:** Jianxun Geng, Liang Chen, Xiaomin Yang, Lili Geng, Jiangyan Duan, Meiling Wang

**Affiliations:** 1 LinFen Vocational and Technical College, Linfen, China; 2 College of Plant Protection, Shanxi Agricultural University, Taigu, China; 3 Institute of Plant Protection, Chinese Academy of Agricultural Sciences, Beiling, China

**Keywords:** Pear tree, Rhizobacteria, 16S rRNA gene sequencing, Antagonistic bacteria, Ecological adaptation

## Abstract

Rhizosphere bacteria play a crucial role in promoting plant health and development. A full understanding of the bacterial communities in rhizosphere soil and their relationship with those in bulk soil is important for supporting plant growth. Some beneficial bacteria are recruited to the root-zone when plants experience different stresses. However, it is unknown whether the rhizosphere of pear trees enriches some beneficial microorganisms that can resist pathogen infections in natural ecosystems. In this study, we found a higher proportion of antagonistic strains in the rhizosphere of pear trees compared to bulk soil under natural growth conditions. By deep sequencing the V3 and V4 hypervariable regions of 16S rRNA genes, we characterized the bacterial communities in the rhizosphere soil of ‘Yuluxiang’ pear trees and the adjacent bulk soil. Our analysis revealed that the diversity of the bacterial community in the rhizosphere soil was lower than that in the bulk soil, but their compositions differed between the two soil types. We found that 12 phyla, 97 families and 130 genera contributed to these differences. Proteobacteria was the dominant phylum and its relative abundance in the rhizosphere soil was significantly higher than that in the bulk soil. Notably, the two genera, *Bacillus* and *Pseudomonas*, were more abundant in the rhizosphere soil. Compared with the bulk soil, the pear rhizosphere soil existed a higher proportion of beneficial bacteria with antagonistic activities against *Fusarium oxysporum*. These findings indicated that the pear tree rhizosphere can selectively assemble beneficial bacteria with specific antagonistic activities to address threats from pathogens. The distinct bacterial community structure in the rhizosphere of plants reflects a spontaneous ecological adaptation mechanism of plants to their environment.

## Introduction

In the process of evolution, different plants have developed various defense mechanisms to cope with different environmental stresses, such as temperature, salinity, drought, pests and pathogens. The rhizosphere of plant, a special ecological niche on plant, has gradually attracted attention in recent years due to the diversity functions of the bacterial communities colonizing it. The rhizosphere was initially delineated as a zone of heightened bacterial activity in the soil adjacent to the plant roots (Lynch & Whipps, 1990) that has different interactions with plants. The interaction modes between different plants and rhizosphere bacteria can be divided into two main categories. Some rhizosphere bacteria are beneficial bacteria for plants (Berendsen et al., 2018). They can promote plant growth, antagonize and compete with pathogens and induce plant resistance, such as *Bacillus* (Xia et al., 2024), *Pseudomonas* (Susanne et al., 2018), *Arthrobacter* (Xu et al., 2021). Some bacteria are potential soil-borne pathogens, leading to plant yield reduction and so on (Bains et al., 2009). Blom et al. (2011) tested the effects of volatiles produced by 42 strains of plant rhizosphere bacteria on plant growth, and found that in addition to significantly increasing plant biomass, these volatiles could also lead to plant death. Xu et al. (2021) found that the relative abundance of bacteria with potential pathogenic phenotypes was higher in the rhizosphere of peanut. Therefore, plants need to balance their rhizosphere microbial ecosystem through the interaction with different bacteria.

The rhizosphere region harbors a large number of microorganisms that have different biological functions in the progress of the interactions with host plants (Xu et al., 2021; Egamberdieva et al., 2008). The rhizospheres of many plants, such as wheat, bean, kiwifruit and banana selected beneficial microorganisms from the bulk soil to address biotic and abiotic stresses (Ling, Wang & Kuzyakov, 2022; Jamil et al., 2022; Cardoso et al., 2021; Yin et al., 2021; Yan et al., 2023). Within the rhizosphere of *Capsicum annuum*, a microbiome with the ability of promoting plant photosynthetic performance and biomass synthesis was enriched to mitigate drought effects (Rodriguez et al., 2008). Some microbiome with the ability of promoting plant growth and nutrient assimilation were enriched in sorghum and peanut rhizospheres (Xu et al., 2021; Alsabri et al., 2020). Yin et al. (2021) found that successive wheat plantings and application of pathogen could specifically accumulate a group of beneficial microbes with antagonistic activities. Yan et al. (2023) isolated a large number of antagonistic bacteria from the kiwifruit rhizosphere, such as *Bacillus* and *Acinetobacter*, which have antagonistic effects against *Pseudomonas syringae* pv. *actinidiae*. Enriched *Flavobacterium* in the rhizosphere of resistant tomato plants could effectively suppress the occurrence of *Ralstonia solanacearum* in susceptible plants (Kwak et al., 2018). In addition, Ling, Wang & Kuzyakov (2022) analyzed the microbial composition and functions of the rhizosphere soil and the bulk soil from different ecosystems, including farmland crops, herbaceous plants, forest trees and the main differences were generalized. The rhizosphere selected some microorganisms from the bulk soil to function as a seed bank, but the bacterial diversity was reduced by 0.9%−5.3%. Compared with the bulk soil, the abundances of Bacteroidetes, Proteobacteria and Actinobacteria increased, whereas those of Nitrospirae, Gemmatimonadetes, Acidobacteria and Chloroflexi decreased in the rhizosphere soil. All bacterial metabolic and ecologically relevant functional predictions showed that genes involved organic compound conversion, nitrogen fixation, denitrification and pathogenesis were enriched in rhizosphere soil, while genes involved in nitrification were decreased. As the rhizosphere was a special ecological zone connecting plant roots and soil and played a crucial role in the dynamics of soil C and N dynamics, it hosted diverse bacteria capable of performing C and N transformation-related functions (Ling, Wang & Kuzyakov, 2022). As an indispensable fruit in daily life, it remains unknown whether the rhizosphere of pear trees can enrich certain beneficial microorganisms when growing naturally.

Pears are one of the most important fruits in the world and are widely cultivated in many countries, including China, the United States, Spain *etc*. In recent years, as people’s living quality have risen, the demand for high-quality pears has steadily increased. To fulfill the expectations of consumers, some excellent pear tree varieties have emerged (Wu et al., 2013; Zhang et al., 2010). In particular, ‘Yuluxiang’ is a high-quality pear tree variety with high-sugar-content, crisp and juicy. It is primarily cultivated in the northern part of China. However, in recent years, the spread of various fruit tree diseases has seriously affected the quality and economic benefits of fruits. Fruit tree diseases mainly include fruit diseases, stem diseases, root diseases, and leaf diseases. For example, in the case of pear tree disease caused by *Potebniamyces pyri* (leading to Phacidiopycnis rot of pear trees), when fruits were infected in the orchard, they typically develop the symptoms during storage. Meanwhile, this can also trigger cankers, dead bark and twig branches of pear trees (Xiao & Boal, 2005). In addition, *Rosellinia necatrix* caused a variety of diseases in pear trees, ranging from rotting of roots, yellowing of leaves and wilting to the eventual death of the trees (Sawant et al., 2021). As a pathogenic fungus, the genus *Fusarium* had the capacity to induce wilt and rot diseases, primarily affecting the roots and stem bases of a variety of fruit trees, including pears (Li et al., 2024), apples (Tang et al., 2022), and bananas (Ma et al., 2023). The rhizosphere of plants could enrich with a large number of biocontrol bacteria that can directly or indirectly inhibit the infection of plant pathogens. Therefore, clarifying the taxonomic and functions of the rhizosphere microorganisms of pear trees, and how they vary from those of the bulk soil microbiome plays an important role in exploring rhizosphere biocontrol resources. Herein, the methods of the 16S rRNA gene sequencing, integrated taxonomic data and functional analysis were used to analyze the microbial communities in the pear rhizosphere and bulk soils. Furthermore, the distribution ratio of antagonistic strains in the rhizosphere and bulk soils was evaluated with *F. oxysporum* as the control targets.

## Materials & Methods

### Study area and site selection

**Figure 1:**
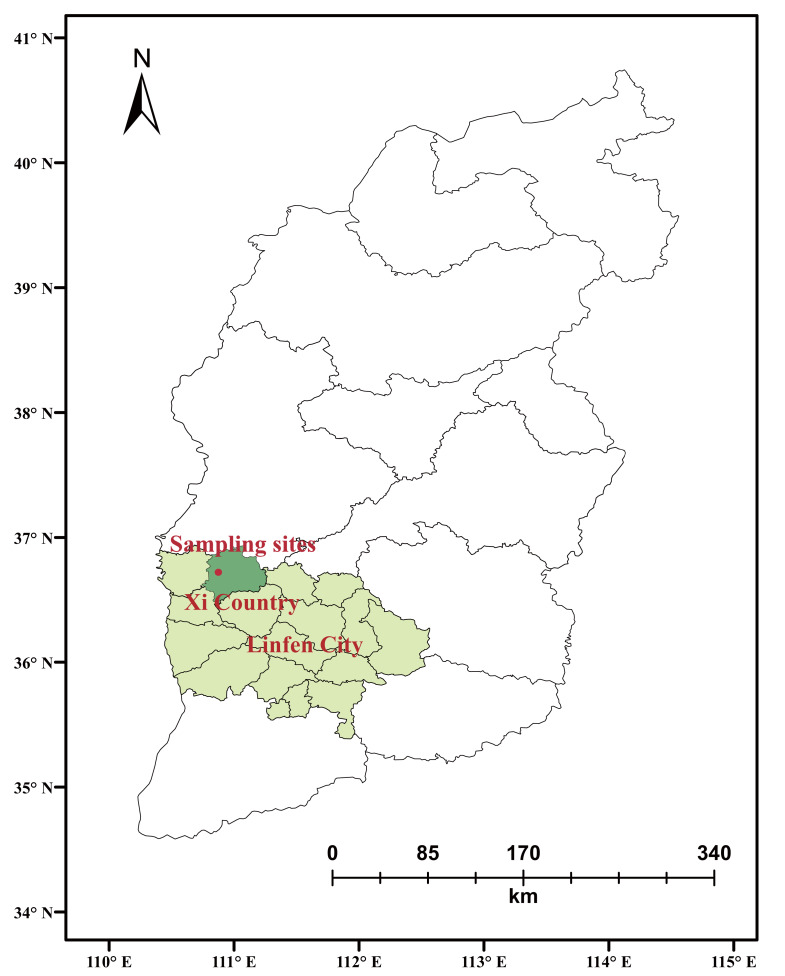
Location of the soil site.

This study area was located in Xi County, Linfen City, Shanxi Province, northwest China (110°44′–111°15′E, 36°27′–36°85′N) (Fig. 1). This region has a planting history of ‘Yuluxiang’ pear trees. In 1999, it was named “Hometown of Chinese Golden Pears” by the Ministry of Agriculture of China. The elevation of this area ranges from 762.5 m to 1,300 m. This area is a temperate continental monsoon climate, with the annual average temperature of 9.6 °C and the average temperature in July is 23.0 °C. The annual average rainfall is 474.2 mm. The main soil types are purple-red clay and brownish-yellow sandy soil (Table 1).

**Table 1:** Characteristics of the soil site.

Characteristics	Soil sample
Elevation	1,100 m
Longitude	110°96′E
Latitude	36°71′N
Slope	15°
Soil type	Purple-red clay and brownish-yellow sandy soil

### Soil sampling

In this experiment, all soil samples were collected from ‘Yuluxiang’ pear orchard. Two soil types were pear tree rhizosphere soil and the bulk soil. A total of seven rhizosphere soil samples (YLX 1-7) and six bulk soil samples (YLX 1-6Y) were collected. Each rhizosphere soil sample was taken from the rhizosphere of an individual pear tree in the pear orchard. These pear trees were healthy, high-yielding, and showed no disease symptoms. The specific method for collecting rhizosphere soil followed the description of Geng et al. (2018). Pear tree root segments were cut and gently shaken to remove some of the loose soil attached to their surface, and then placed the root segments into a sterile centrifuge tube (50 mL) containing sterile phosphate-buffered saline (PBS) buffer solution. After soaking for 15 min, the solution was vortexed for 30 s. Next, removing the roots, the remaining soil suspension was centrifuged at 3,200 ×g for 15 min. The precipitate was gently resuspended in a small amount of sterile PBS buffer, and subsequently centrifuged once more at 5,000 ×g for a duration of 15 min. And the precipitate obtained was the rhizosphere soil sample. Bulk soil was collected from 10 cm below the surface of the field ridges in the pear orchard mentioned above and the preparation method of the bulk soil samples were carried out as mentioned above. Then the sample was divided into two parts: some was used for the preparation of sequencing samples, and the other part was used for the subsequent isolation of strains.

### The 16S rRNA gene sequencing

A total of 13 soil samples were obtained for the two treatments, comprising seven rhizosphere soil samples and six bulk soil samples. For each soil sample, 0.5 grams of soil was utilized to isolate and extract the total DNA. The extraction of the total DNA of soil was used the PowerSoil^®^ DNA Isolation Kit following the instructions.

The sequencing of microbial diversity was based on the Illumina NovaSeq sequencing platform. By adopting the paired-end sequencing method, primers 338 F and 806 R with sequencing adapters were used to amplify the 16S rRNA (V3 + V4) conserved region (Table S1). The PCR amplification products underwent purification, quantification, normalization, and were subsequently assembled into a sequencing library. After passing the quality inspection, the library was sequenced using the advanced Illumina NovaSeq 6,000 platform. The raw data files generated from high-throughput sequencing process were meticulously converted into raw sequencing reads through base recognition analysis. All sequencing results were deposited in the Sequence Read Archive (SRA) database (PRJNA1213351) with the accession numbers were SRR32103971 to SRR32103983.

### The analysis of 16S rRNA sequencing data

The raw sequences acquired from sequencing underwent filtering using Trimmomatic v0.33 software (Bolger, Marc & Bjoern, 2014), primer sequences were identified and subsequently removed with the software of Cutadapt 1.9.1 (Martin, 2011), and the paired-end sequences were integrated using Usearch v10 (Edgar, 2013) following the method of Geng et al. (2018). In this study, the UCHIME v4.2 software (Edgar et al., 2011) was employed to remove chimeric sequences. Finally, these high-quality reads were parted into different operational taxonomic units (OTUs) (sequence similarity of 97% was defined as an OTU) by using de novo clustering method. Species classification was obtained using the Silva reference database based on the bayesian classifier.

The alpha diversity index of different samples was evaluated by using QIIME2 2020.6 software (Bolyen et al., 2019) and all data differences were evaluated by the method of the Mann–Whitney U test. Analysis of similarities (ANOSIM) based on Bray–Curtis analysis, principal coordinate analysis (PCoA) and non-metricmulti-dimensional scaling (NMDS) were done by using R software (version 3.4.0; R Core Team, 2024). The relative abundance differences between the rhizosphere soil and bulk soil at different classification levels were assessed by using the Wilcoxon rank-sum test in BMKCloud (https://www.biocloud.net/). Co-occurrence networks of different treatments were done by online MENA pipeline (Zhou et al., 2010), and the graphics were exported by using the Gephi (version 0.9.7) (Bastian, Heymann & Jacomy, 2009).

### Culture collection of rhizosphere bacteria

The pear tree root segments were collected and immersed in sterile PBS buffer solution and vortexed as described above. Next, removing the roots, the remaining soil suspension was centrifuged at 3,200 ×g for a duration of 15 min. The precipitate was resuspended in five milliliter PBS buffer. Bulk soil samples were treated follow the same method. Then, the cell suspensions were diluted 10-fold with PBS buffer, plated on the Luria-Bertani (LB) solid medium plates and incubated for 48 h at 30 °C. Ninety-six colonies of different morphologies were randomly selected and each colony was transferred into a different well of a 96-well plate containing 500 µL of LB medium. All colonies were kept at 30 °C at 220 rpm/min for 48 h.

### The analysis of antagonism activity

The antagonistic effects between the above-mentioned different strains and plant pathogens were analyzed using a plate confrontation assay. In this experiment, a total of seven plant pathogenic fungi were tested, including *F. oxysporum*, *F. acuminatum*, *Botrytis cinerea*, *F. dermatis*, *Sclerotinia sclerotiorum*, *Exserohilum turcicum* and *F. graminearum*. A 6.0-mm pathogen mycelial disk was placed upside down at the center of a PDA solid medium plate (15 cm). Twelve sterile filter papers were evenly placed at a distance of 3.0 cm from the center of the plate. Subsequently, 5.0 µL of each strain’s culture broth was dropped onto the filter papers. Finally, these plates were placed at 25 °C for 10–20 d, and the antibacterial effects were recorded.

## Results

### Illumina NovaSeq data

To investigate the variations in microbial community structure and functionality in pear tree rhizosphere soil and bulk soil under normal growth conditions, we collected and characterized the rhizosphere soil (seven samples, YLX 1-7) and bulk soil (six samples, YLX 1-6Y) from ‘Yuluxiang’ pear trees. These samples underwent sequencing of the V3-V4 region of the 16S rRNA genes for detailed analysis. Sequencing process yielded a total of 2,753,245 raw reads. Subsequent filtering refined this dataset to 1,725,035 clean reads across thirteen biological samples (Table S2). Each sample had an average read length of 422 bp (Table S2). From the rhizosphere soil and bulk soil, averages of 949 OTUs and 995 OTUs were identified, respectively, as outlined in Table S2. The rarefaction curve indicated that the number of OTUs for each sample reached a plateau, and it suggested that the sequencing data had adequate depth (Fig. S1).

### Alpha diversity-based comparison in pear rhizosphere and bulk soils

The alpha diversity of the two groups was evaluated by four indices (Table 2, Table S3). The Abundance-based Coverage Estimator (ACE) index, Chao1 index, Simpson index and Shannon index for rhizosphere soil were 951.246 ± 93.162, 949.578 ± 85.129, 0.942 ± 0.009 and 6.946 ± 0.349, while those for bulk soil were 996.016 ± 85.293, 994.586 ± 85.129, 0.966 ± 0.009 and 7.792 ± 0.349, respectively. Compared to the Simpson index and Shannon index value of the bulk soil, the rhizosphere soil group was significantly lower. No statistically significant differences were observed in the ACE index and Chao1 index between two groups. These results indicated that two treatments had comparable bacterial richness, but the bulk soil had a greater species diversity.

**Table 2:** Alpha diversity indices of pear rhizosphere and bulk soils.

Diversity index	Rhizosphere soil (Mean ± SD)	Bulk soil (Mean ± SD)
ACE	951.246 ± 93.162 a	996.016 ± 85.293 a
Chao1	949.578 ± 85.129 a	994.586 ± 85.129 a
Simpson	0.942 ± 0.009 b	0.966 ± 0.009 a
Shannon	6.946 ± 0.349 b	7.792 ± 0.349 a

**Notes.**

Different lowercase letters mean significant differences between the two groups (the Mann–Whitney U test, *p* < 0.01).

### Beta diversity-based comparison in pear rhizosphere and bulk soils

**Figure 2:**
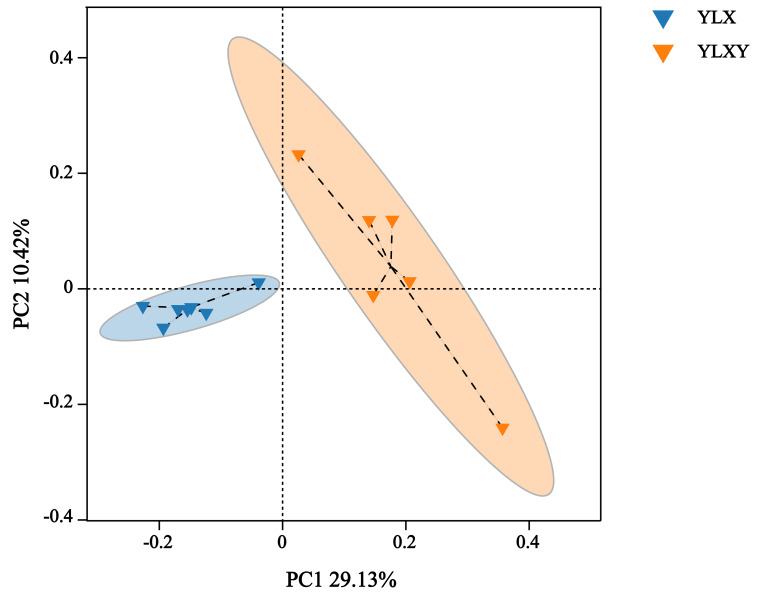
The PCoA analysis between different groups by Bray–Curtis method. YLX, pear rhizosphere soil; YLXY, bulk soil.

PCoA, NMDS and ANOSIM were used to analyze the differences in the bacterial community structure between the pear rhizosphere soil and bulk soil. The first principal coordinate (PC1, 29.13%) suggested that the bacterial communities in the rhizosphere soil differed significantly from those in the bulk soil. Additionally, PC2 also showed 10.42% inter-sample variance (Fig. 2). The NMDS result showed that the stress value was 0.062 (Fig. S2). Furthermore, the ANOSIM analysis based on effective OTUs reconfirmed that the bacterial compositions of the pear rhizosphere and bulk soils were significantly different, with an R-value of 0.731 and a *p*-value of 0.002 (Fig. S3).

### Taxonomy-based analysis of pear rhizosphere and bulk soils

**Figure 3:**
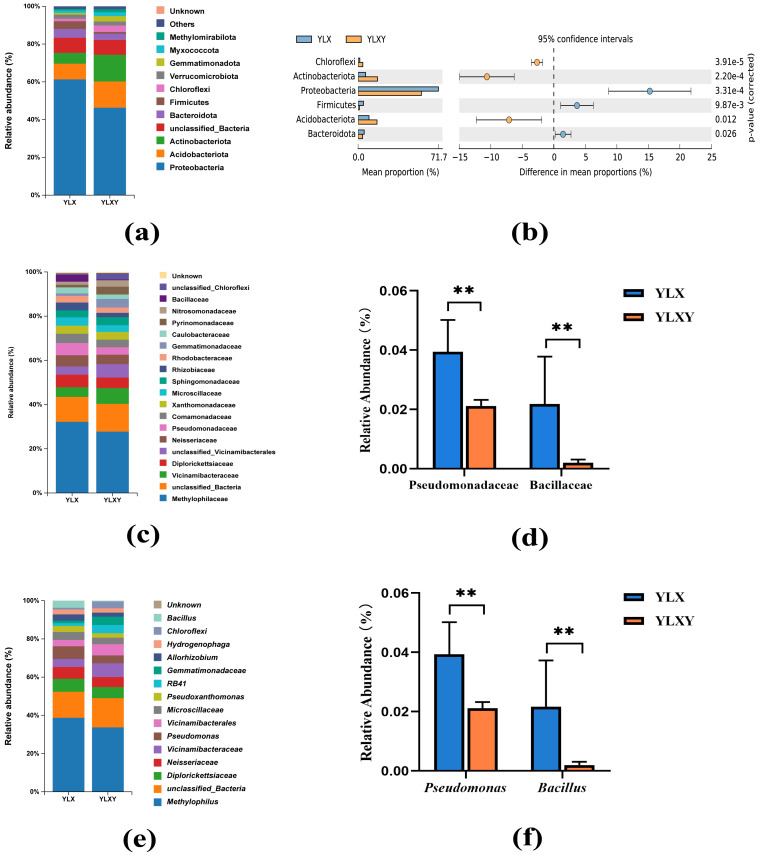
The relative abundance of the pear rhizosphere and bulk soil microbiome compositions and the difference in bacteria. (A) Microbiome composition at the phylum level. (B) The significant differences between the top six phyla in the pear rhizosphere and bulk soils. (C) Microbiome composition at the family level. (D) The relative abundance of Pseudomonadaceae and Bacillaceae in the pear rhizosphere and bulk soils. (E) Microbiome composition at the genus level. (F) The relative abundance of *Pseudomonas* and *Bacillus* in the pear rhizosphere and bulk soils. The significant differences between different phyla/families/genera were analyzed by Wilcoxon rank-sum test (** *p* < 0.01). YLX, pear rhizosphere soil; YLXY, bulk soil.

A total of 45 phyla, 544 families and 1035 genera were found in two treatments. The relative abundance of the two groups in the taxon-based evaluations was showed in Tables S4–S6. Twelve phyla contributed to the differences in the rhizosphere soil and the relative abundance of six phyla was > 1%, among which Proteobacteria was the dominant phylum and contributed over 60% of all found phyla (Figs. 3A–3B and Table S4). Compared with the rhizosphere soil, the relative abundance of Proteobacteria (from 61.24% to 46.30%), Bacteroidota (from 4.68% to 3.29%) and Firmicutes (from 4.07% to 0.90%) decreased, whereas those of Acidobacteriota (from 8.34% to 13.83%), Actinobacteriota (from 5.72% to 14.21%) and Chloroflexi (from 1.33% to 3.46%) increased in the bulk soil (Fig. 3A and Table S4). In total, 97 families and 130 genera contributed to the differences in the bacterial communities of the rhizosphere and bulk soils (Tables S5 and S6). The mean relative abundances of 12 families and 12 genera exceeded 1%. The increase in Pseudomonadaceae (from 2.12% to 3.96%) within Proteobacteria and Bacillaceae (from 0.20% to 2.19%) within Firmicutes in the rhizosphere soil was driven by a set of genera, predominantly *Pseudomonas* (from 2.12% to 3.94%) and *Bacillus* (from 0.20% to 2.16%) (Figs. 3C–3F). Thus, pear rhizosphere soil enriched the Pseudomonadaceae and Bacillaceae.

### Differences in pear rhizosphere soil and bulk soil microbial phenotypes

**Figure 4:**
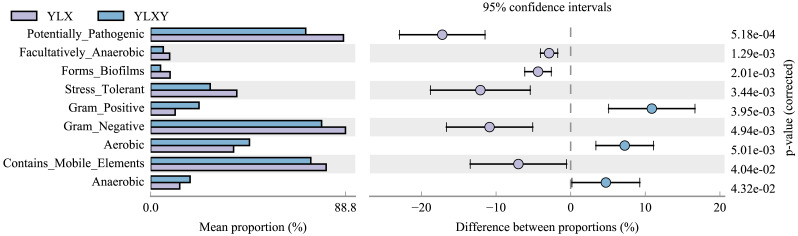
Bacterial phenotypic differences between the pear rhizosphere soil and bulk soil predicted by BugBase analysis. (Student’s *t*-test, *p* < 0.05). YLX, pear rhizosphere soil; YLXY, bulk soil.

BugBase was used to predict the coverage of functional pathways at the biological level within intricate microbiomes, and to provide biologically interpretations of the phenotypes. Herein, this method was used to analyze the phenotypic differences between the pear rhizosphere soil and bulk soil samples based on the 16S rRNA sequence results. The relative abundance of biofilm-forming microorganisms was higher in the pear rhizosphere soil sample (Fig. 4 and Table S7), mainly due to the greater abundance of Comamonadaceae, Methylophilaceae, Pseudomonadaceae, Rhodospirillaceae and Xanthomonadaceae (Fig. S4A). The relative abundance of bacteria with stress-tolerant phenotypes of the pear rhizosphere soil was significantly higher than that in the bulk soil (Fig. 4 and Table S7), which was mainly attributed to the greater abundance of Comamonadaceae, Methylophilaceae, Pseudomonadaceae and Xanthomonadaceae (Fig. S4B). The relative abundance of potential pathogenic bacteria and those with gene functions related to mobile elements were significantly more abundant in the pear rhizosphere soil sample than in the bulk soil (Fig. 4 and Table S7), which were primarily attributed to the greater abundance of taxa belonging to Bacillaceae, Comamonadaceae, Cytophagaceae, Methylophilaceae, Pseudomonadaceae, Rhodospirillaceae and Xanthomonadaceae (Figs. S4C and S4D). In addition, gram-positive, aerobic and anaerobic bacteria were less abundant in the pear rhizosphere soil than in the bulk soil (Fig. 4 and Table S7).

### Differences in pear rhizosphere soil and bulk soil bacterial ecological functions

**Figure 5:**
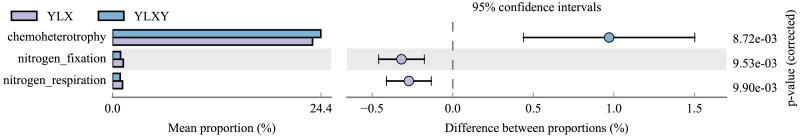
Bacterial ecological functions differences between the pear rhizosphere soil and bulk soil predicted by FAPROTAX. (Student’s *t*-test, *p* < 0.05). YLX, pear rhizosphere soil; YLXY, bulk soil.

FAPROTAX was suitable for annotating and predicting ecological functions of environmental samples. In this study, this tool was used to analyze the functional differences between the pear rhizosphere soil and bulk soil samples based on the 16S rRNA sequence results. The top twelve bacterial functional groups (with mean relative abundances greater than 1%) included chemoheterotrophy, nitrate_reduction, ureolysis, methylotrophy, methanol_oxidation, aerobic_chemoheterotrophy, fermentation, animal_parasites_or_symbionts, nitrogen_fixation, nitrogen_respiration, nitrate_respiration and human_pathogens_all (Table S8). Three of the top twelve functional abundances exhibited obvious differences, among which the relative abundances of nitrogen_fixation and nitrogen_respiration were higher in the pear rhizosphere soil, while the relative abundance of chemoheterotrophy in the bulk soil was higher (Fig. 5).

### Analysis of co-occurrence networks

**Figure 6:**
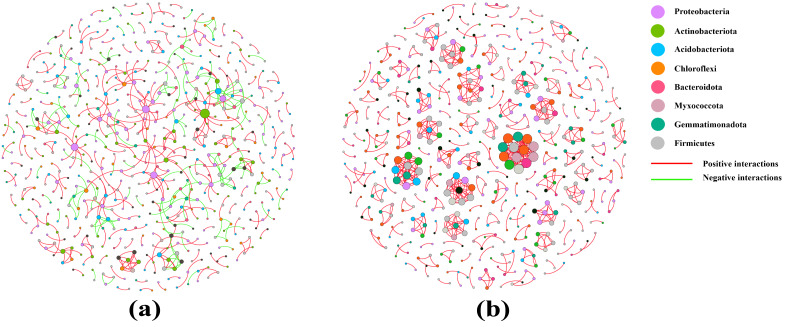
Co-occurrence networks of the rhizosphere soil (A) and the bulk soil (B).

Co-occurrence networks were used to test the complexity of different soil sample microbiomes. Compared with that of the pear rhizosphere soil, the microbiome network modularity of the bulk soil had a highly connected community with higher total edges (718), greater average degree (2.635) and a shorter average path distance (1.000), but the total nodes was 545. Therefore, the pear bulk soil microbiomes had a more complex co-occurrence network than the rhizosphere soil (Fig. 6 and Table 3).

### Analysis of antifungal capabilities of bacteria

**Figure 7:**
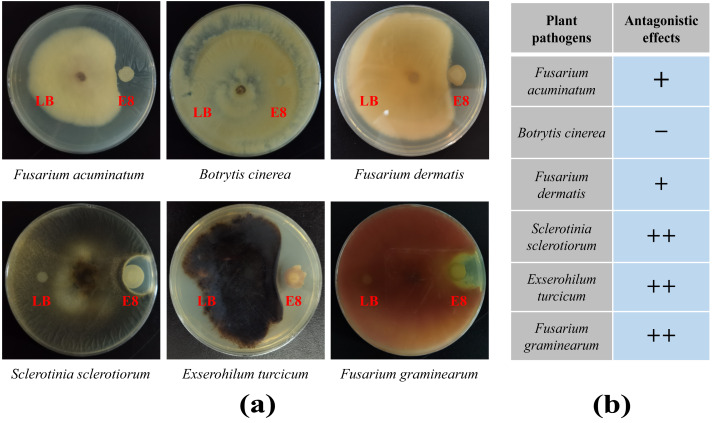
The antifungal effects of E8 colony from rhizosphere soil. (A) Confrontation results of E8 colony against different pathogens. (B) Analysis of the antifungal activities of E8 colony against different pathogens. “+ +” indicates a stronger antifungal effect, “+” indicates a weak antifungal effect, and “−” indicates the absence of any antifungal effect.

To demonstrate the capability of plants to recruit beneficial bacteria, this experiment utilized LB medium to randomly isolate 96 cultivable clones from two groups and employed plate confrontation experiments to analyze their antifungal abilities against *F. oxysporum*. The results showed that a total of 41 colonies in rhizosphere soil exhibited antagonistic effects to varying degrees, accounting for 42.7%. In the bulk soil, 14 colonies showed antagonistic effects, accounting for 14.6%, which was significantly lower than the proportion of antagonistic strains in the rhizosphere soil (Tables S9 and S10). To further verify whether the antibacterial effects of these antagonistic colonies were broad-spectrum, colony of E8 from the rhizosphere soil, which exhibited significant antibacterial activity, was selected to analyze its antifungal abilities against six plant diseases, including *F. acuminatum*, *B. cinerea*, *F. dermatis*, *S. sclerotiorum*, *E. turcicum* and *F. graminearum*. We found that it had antagonistic effects against five of the tested pathogenic fungi but not against *B. cinere* (Fig. 7). These results demonstrated that the rhizosphere of pear trees can recruit biocontrol strains that are beneficial to plants.

**Table 3:** Topological properties of networks.

Network properties	Rhizosphere soil	Bulk soil
Total nodes	655	545
Total links	615	718
Average path distance	3.519	1.000
Average degree	1.878	2.635
Average clustering coefficient	0.170	0.567

## Discussion

The rhizosphere is the soil environment where plant roots conduct their normal vital activities, and where huge amounts of microorganisms colonize. It plays an important role in maintaining plant growth and health. “Rhizosphere microorganisms” is a collective term for microorganisms that inhabit the rhizosphere environment and are influenced by root exudates (Berendsen, Pieterse & Bakker, 2012). Their abundance typically reaches 10^9^ to 10^12^ microorganisms per gram of soil, which is five to 10 times higher than the number of microorganisms found in non-rhizosphere soil (Miransari, 2011; Groleau-Renaud et al., 2000). In the process of gradual evolution, recent studies have found a decreasing and selective trend in the bacterial richness and diversity of rhizosphere microorganisms in real agricultural systems (Ling, Wang & Kuzyakov, 2022). Wei et al. (2023) found that bacterial alpha diversity indices were lower in the rhizosphere soil of Chinese cabbage than that in the bulk soil. These results showed a similar trend to those findings in our study, confirming that pear rhizosphere soil and bulk soil had comparable bacterial richness and that the bulk soil had greater species diversity (Table 2, Table S3). Co-occurrence network diagrams can be utilized to study the interaction relationships among a large number of microbial species (Rottjers & Faust, 2018). Researches had revealed that in short-term farming systems, the network relationships in the rhizosphere soil of Chinese cabbage and soybean plants were simpler compared to those in the bulk soil (Wei et al., 2023; Mendes et al., 2014). In this study, the co-occurrence network in the rhizosphere soil of pear displayed a weaker structure than that in the bulk soil. The co-occurrence networks of rhizosphere soil encompassed both positive interactions (422) and negative interactions (193), whereas the bulk soil exhibited almost exclusively positive interactions. Consequently, the interactions among bacteria in bulk soil primarily were mutualistic and synergistic relationships, while the rhizosphere soil contained not only symbiotic bacterial communities but also certain bacterial communities with antagonistic, competitive and parasitic relationships. Furthermore, both the co-occurrence networks of rhizosphere soil and bulk soil contained multiple modular interaction units. In the bulk soil, these interactions involved multiple phyla, whereas in the less modular rhizosphere soil, the interactions were mostly centered around Proteobacteria (Fig. 6, Table 3). These results once again demonstrated that the bacterial community diversity in the rhizosphere soil of pear trees was lower than that in the bulk soil, and that Proteobacteria was the dominant phylum.

The diversity of bacterial communities in the rhizosphere soil was lower than that of the bacterial communities in the bulk soil. However, their compositions differed between the two soil types. The analysis results of PCoA, NMDS and ANOSIM showed that their microbial community structure and composition differed between two types of soils (Fig. 2, Figs. S2 and S3). The rhizosphere soil was characterized by Proteobacteria (61.2%), Acidobacteriota (8.3%), Actinobacteriota (5.7%), Bacteroidota (4.7%) and Firmicutes (4.1%). In contrast, Proteobacteria (46.3%), Actinobacteriota (14.2%), Acidobacteriota (13.8%), Chloroflexi (3.5%) and Bacteroidota (3.3%) were the dominant phyla in the bulk soil. Among these, compared to the bulk soil, the relative abundance of Proteobacteria and Bacteroidota were higher in the rhizosphere soil (Fig. 3A and Table S4). Ling, Wang & Kuzyakov (2022) found that Proteobacteria and Bacteroidota were capable of rapid growth in carbon-rich soil environments, and had higher metabolic activity and reproductive capacity, and were more abundant in plant rhizosphere soil than in bulk soil. Therefore, these results indicated that the rhizosphere soil of pear trees had a higher metabolic activity and nutrient availability than the bulk soil. This spontaneous environmental adaptation behavior of plants not only promoted their own growth but also had the potential to benefit soil health.

Factors influencing the structure and composition of microbial communities in the plant rhizosphere were relatively complex. The physicochemical properties of soil were the primary determinants, followed by the environmental conditions, host genotypes, and soil nutrient status (Stopnisek & Shade, 2021; Lundberg et al., 2012; Yeoh et al., 2017). Moreover, plants often developed their defense mechanisms against different pathogens by selective stimulation and support from antagonistic bacteria. *Bacillus* strains, including *B. subtilis* and *B. cereus etc*., represented the most promising biological control agents currently available. They primarily prevented plant diseases by producing antibacterial substances and inducing systemic resistance in plants (Jiang et al., 2016; Vlamakis et al., 2013). *B. thuringiensis*, a well-known insect pathogen, was also gradually being recognized for its potential in the biological control of plant diseases (Wang et al., 2020). Different *Pseudomonas* species could also act as beneficial to plants and insect-pathogenic roles (Vesga et al., 2020). Shen et al. (2015) found that the *Bacillus* and *Pseudomonas* genera were enriched in healthy soil of banana. In our study, the relative abundance of *Bacillus* and *Pseudomonas* were significantly increased in pear rhizosphere soil (Figs. 3C–3F). Moreover, BugBase analysis showed biofilm-forming, stress-tolerant bacteria related to disease resistance were significant increased in the pear rhizosphere soil sample (Fig. 4). However, the potential pathogenic microorganisms were also enriched in the rhizosphere (Fig. 4), which also found in other researches (Xu et al., 2021; Wei et al., 2023). The family Xanthomonadaceae, belonging to the phylum Proteobacteria, was predominantly composed of plant pathogens, including multiple pathogenic species labeled as *Xanthomonas*, *Xylella* and *Stenotrophomonas*. These bacteria primarily adhered to the surface of host tissues through the production of exopolysaccharides, lipopolysaccharides, and proteineous structures, thereby causing various plant diseases such as citrus canker (Mhedbi-Hajri, Jacques & Koebnik, 2011; Shahbaz et al., 2022). Notably, a minority of strains could also function as plant probiotics (Rat et al., 2021; Stork, Kim & Mudgett, 2015). The increase in the abundance of biofilm-forming, stress-tolerant and potential pathogenic phenotype-related bacteria in the pear rhizosphere soil was all associated with the rise in the abundance of Xanthomonadaceae (Fig. S4). Therefore, plants must balance their rhizosphere microbial ecosystem by different defense mechanisms.

The extensive use of chemical bactericides and other pesticides had led to issues such as pesticide residues and resistance. Therefore, there is an urgent need to explore new microbial strain resources with strong disease resistance and broad antibacterial spectrums. In our study, the analysis results of the plate confrontation experiment between bacteria collected from different ecological niches and *F. oxysporum* revealed that the rhizosphere soil harbored numerous bacteria with antagonistic effects (Fig. 7, Tables S9 and S10). According to literature reports, over 80% of the clones directly isolated contain multiple OTUs (Xu et al., 2024). Therefore, we further isolated bacteria from the E8 colony and conducted 16S rDNA full-length sequence analysis. The analysis results showed that the presence of strains belonging to the *Bacillus* and *Pantoea*, and further confirmed that these strains had well antagonistic effects against *F. dermatis*, *S. sclerotiorum* and *F. graminearum*. Notably, it was reconfirmed that *Bacillus* was enriched in the rhizosphere soil. In summary, these results indicated that the plant rhizosphere could recruit specific biocontrol strains, such as *Bacillus*. Previous research by our team had found that after insect feeding, the rhizosphere of plants enriched some strains possessing insecticidal activity (Xu et al., 2024). Herein, a high abundance of *Bacillus* was found in the rhizosphere soil of pear trees (Fig. 3F). Notably, *B. thuringiensis*, belonging to the *Bacillus* genus, was both an insect pathogen and capable of enhancing plant disease resistance (Jouzani, Valijanian & Sharafi, 2017; Wang et al., 2023). Therefore, further analysis should be conducted to investigate the potential of bacterial genera enriched in the rhizosphere of pear trees for controlling pear pests, such as *Grapholitha molesta*. It is expected to isolate the biocontrol strain resources from the rhizosphere of pear trees with the potential for pest and disease management.

## Conclusions

Taken together, our results indicated that the diversity of bacteria communities in the bulk soil was higher than that in the rhizosphere soil, but their compositions differed between two types of soil. The pear rhizosphere soil exhibited a significantly higher relative abundance of bacterial phyla including Proteobacteria, Bacteroidota and Firmicutes, while Acidobacteriota, Actinobacteriota, and Chloroflexi were more prevalent in the bulk soil. The genera of rhizosphere bacteria, specifically *Methylophilus*, *Diplorickettsiaceae*, *Pseudomonas*, *Neisseriaceae*, *Microscillaceae* and *Bacillus*, were significantly higher in the pear rhizosphere. Among these genera, *Bacillus* and *Pseudomonas* were typical biocontrol strains. The relative abundance of bacteria with biofilm-forming, stress-tolerant and potential pathogenic phenotypes was significantly higher than that in the bulk soil. The FAPROTAX analysis showed the ecological functions of the rhizosphere soil were mainly affected by chemoheterotrophy, nitrogen_fixation and nitrogen_respiration. Moreover, the rhizosphere soil harbored a higher proportion of antagonistic strains. The differences in the bacterial community between the rhizosphere soil and the bulk soil provided an efficient way to exploring beneficial bacterial resources.

##  Supplemental Information

10.7717/peerj.20627/supp-1 Supplemental Information 1 Supplemental Tables

10.7717/peerj.20627/supp-2 Supplemental Information 2 Supplemental Figures

## Additional Information and Declarations

### Data Availability

The following information was supplied regarding data availability: The data are available in the Sequence Read Archive database (NCBI): PRJNA1213351; SRR32103971 to SRR32103983.
