# Supplementary material for: Characteristics of rhizosphere and bulk soil microbial communities in pear trees
Source: PeerJ. 2026 Jan 15;14:e20627. doi: 10.7717/peerj.20627 (PMC12812276; doi:10.7717/peerj.20627)
Supplement: Supplemental Information 2 [file peerj-14-20627-s002.docx]

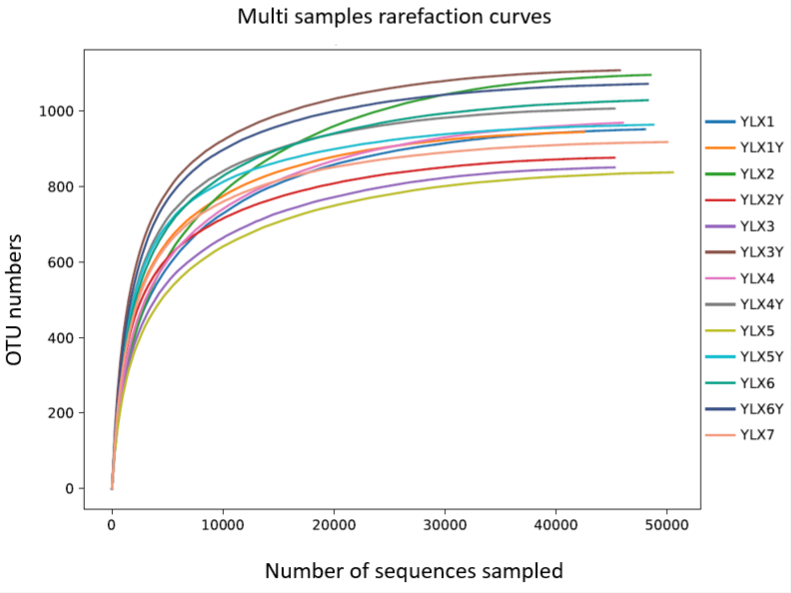


**Figure S1.** Rarefaction curve of 16S rRNA gene sequencing of the rhizosphere and bulk soils. YLX: pear rhizosphere soil; YLXY: bulk soil.


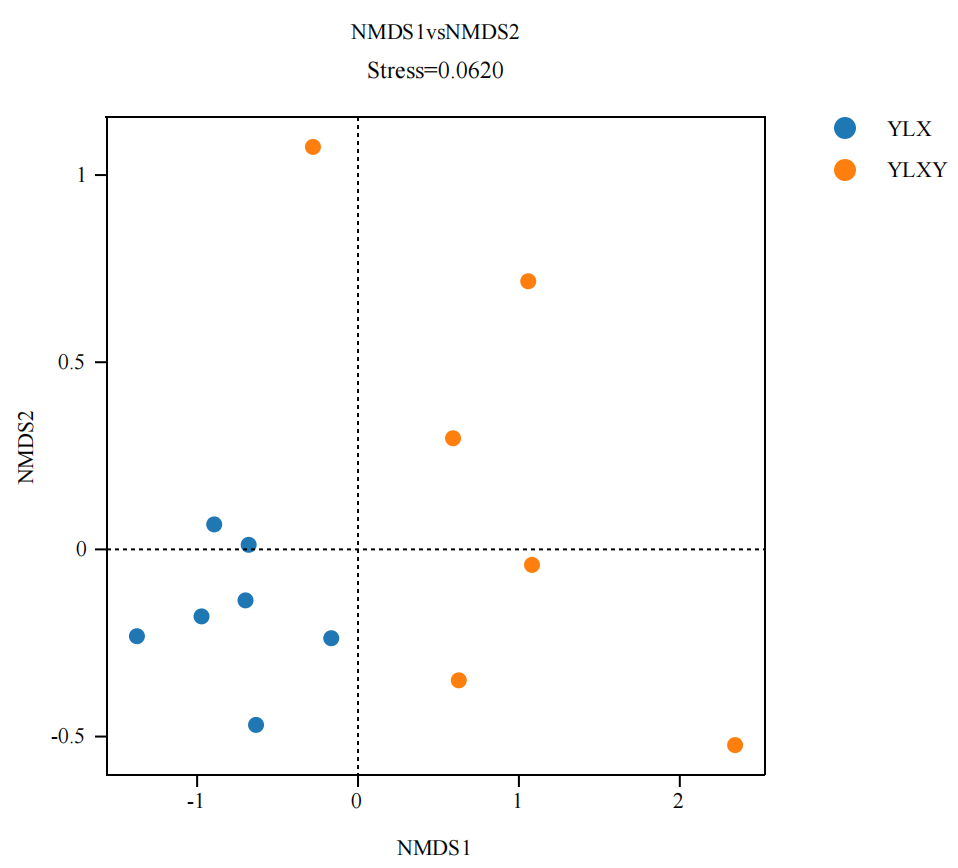


**Figure S2.** The NMDS analysis between different groups by Bray-Curtis method. YLX: pear rhizosphere soil; YLXY: bulk soil.


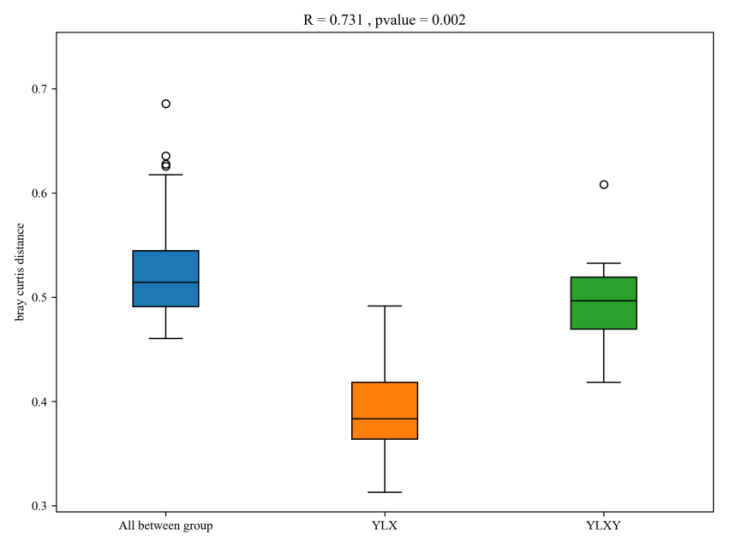


**Figure S3.** The ANOSIM-based effective OTUs between different groups by Bray-Curtis method. YLX: pear rhizosphere soil; YLXY: bulk soil.


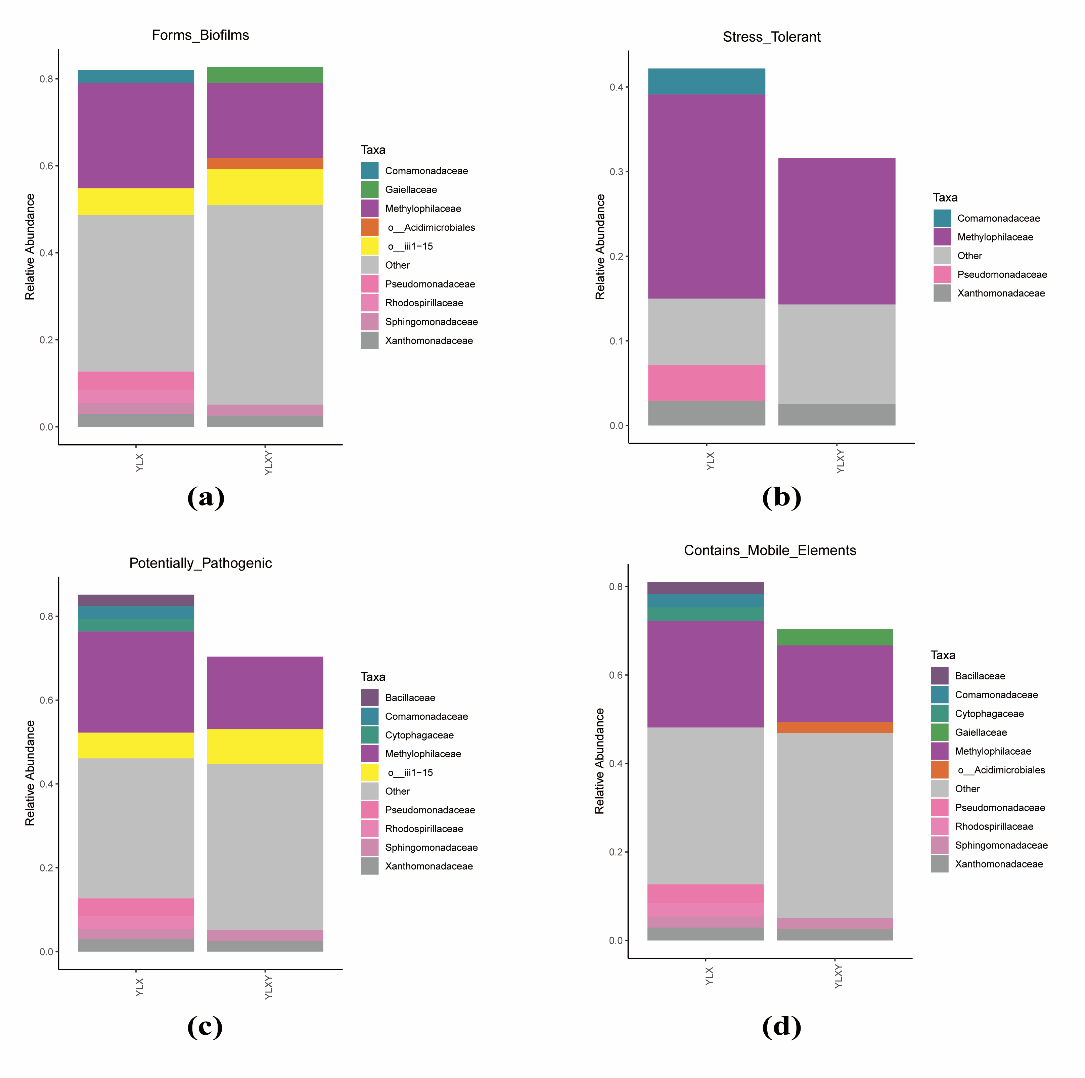


**Figure S4.** The relative abundances of family with phenotypes related to a) biofilm formation, b) stress tolerance, c) potential pathogenicity and d) mobile elements. YLX: pear rhizosphere soil; YLXY: bulk soil.
